# Mirtazapine Induces Lipocalin-Type Prostaglandin D Synthase Expression in Brain Pericytes

**DOI:** 10.3390/biom16070945

**Published:** 2026-06-24

**Authors:** Aya Narita, Akiko Nakano-Doi, Ryo Nishiyama, Toshinori Sawano, Kazuaki Fukushima, Tomohiro Matsuyama, Takayuki Nakagomi

**Affiliations:** 1 Institute for Advanced Medical Sciences, Hyogo Medical University, 1-1 Mukogawacho, Nishinomiya 663-8501, Japan; nari1119@hyo-med.ac.jp (A.N.); nakano@hyo-med.ac.jp (A.N.-D.); ri-nishiyama@hyo-med.ac.jp (R.N.); 2 Department of Therapeutic Progress in Brain Diseases, Hyogo Medical University, 1-1 Mukogawacho, Nishinomiya 663-8501, Japan; tomohiro@hyo-med.ac.jp; 3 Research Center for Advanced Medical Science, Dokkyo Medical University, 880 Kitakobayashi, Mibu-machi, Shimotsuga-gun, Tochigi 321-0293, Japan; t-sawano@dokkyomed.ac.jp; 4 Department of Biomedical Sciences, Ritsumeikan University, 1-1-1 Nojihigashi, Kusatsu 525-8577, Japan; 5 Department of Chemistry, Hyogo Medical University, 1-1 Mukogawacho, Nishinomiya 663-8501, Japan; ka-fukushima@hyo-med.ac.jp

**Keywords:** lipocalin-type prostaglandin D synthase, brain pericytes, waste scavenger, chemical compounds, mirtazapine, cerebrovascular disease, Alzheimer’s disease, sleep disorder, glymphatic system’s perivascular tunnels

## Abstract

The brain maintains homeostasis partially by scavenging waste products. Failure of this function is closely associated with the onset and pathogenesis of various brain diseases, such as Alzheimer’s disease, sleep disorder, and the delay of the reparative process after brain injuries. We recently demonstrated that brain pericytes (BPCs) are sources of lipocalin-type prostaglandin D synthase (L-PGDS), a waste scavenger, in the brain. Based on the above, chemical compounds which promote L-PGDS production could have potential against brain diseases, such as dementia, sleep disorders, and brain injuries. However, the specific chemical compounds that may enhance L-PGDS production in BPCs have not yet been identified. In this study, we explored 158 chemical compounds from FDA-approved drug libraries with these activities. qPCR analysis showed that mirtazapine (MTZ), a noradrenergic and specific serotonergic antidepressant, can increase L-PGDS expression in BPCs as well as in mouse- (m-BPCs) and human-derived BPCs (h-BPCs) in a dose-dependent manner. Since L-PGDS is a secretory protein, m-BPCs and h-BPCs were treated with various MTZ doses and L-PGDS levels in the culture supernatant were investigated. Western blot analysis showed that L-PGDS levels were significantly increased in a dose-dependent manner in both cell types, indicating that MTZ promoted L-PGDS secretion from m-BPCs and h-BPCs. Thus, MTZ may have the potential to be applied as drug repositioning for various brain diseases other than depression by activating L-PGDS production in BPCs, highlighting the importance of BPCs as the source to maintain brain homeostasis.

## 1. Introduction

The brain can maintain homeostasis partly by scavenging waste products [1]. If this process fails, it causes accumulation of waste products, such as amyloid-β (Aβ) and Tau, related to the pathogenesis of brain diseases, such as Alzheimer’s disease (AD) [2,3]. Although the precise mechanism whereby the brain removes waste products remains unclear, there are several possible mechanisms, including phagocytosis by microglia/macrophages (MGs/MΦs) [4] and the glymphatic system [5,6]. Particularly, waste product clearance through the latter is enhanced during sleep [3,7], being disrupted in poor sleepers, which could underlie memory decline [8], indicating a close relationship between the clearance of waste product, sleep, and cognitive impairment. However, the molecules involved in regulating both brain debris clearance and sleep have yet to be identified.

Lipocalin-type prostaglandin D synthase (L-PGDS, or prostaglandin D2 synthase) is a chaperone protein that can scavenge waste products in the brain, such as amyloid-β [9,10] and biliverdin [11] and convert prostaglandin H2 (PGH2) into prostaglandin D2 (PGD2) [12]. Interestingly, PGD2 levels are elevated in pathological brains, such as AD, traumatic brain injury [13], and ischemic stroke [14,15,16,17]. PGD2 binds to PGD2 receptor 1 (DP1) and PGD2 receptor 2 (DP2) [18]. Particularly, DP1 is highly expressed in brain cells, including MGs/MΦs [19,20,21], astrocytes [22], and neurons [23]. In addition, we recently showed that the L-PGDS-PGD2-DP1 axis is activated in injured areas after ischemic stroke and that the L-PGDS-PGD2-CD36 axis is involved in waste product phagocytosis [17]. Although the precise roles of the L-PGDS-PGD2-DP1 axis in the brains remain unclear, previous studies have demonstrated that the L-PGDS-PGD2-DP1 axis is associated with sleep regulation [24], and a recent study showed increased L-PGDS levels in patients with hypersomnia [25]. These results indicate that regulating L-PGDS or its related pathways (L-PGDS–PGD2–DP1 axis) may be effective in treating brain diseases like AD, cerebrovascular disease (CVD), and sleep disorders.

L-PGDS is highly expressed in the leptomeninges, which cover the brain surface [26], and is abundant in the cerebrospinal fluid (CSF) [11,26]. While the exact characteristics of L-PGDS-producing cells in the brain are not fully known, we recently found that L-PGDS is highly expressed in brain pericytes, especially in the leptomeninges [17]. BPCs are a component of the neurovascular unit (NVU), which comprises astrocytes and endothelial cells [27,28]. Although BPCs seem to play a role in maintaining brain function under both normal and pathological conditions, such as by regulating cerebral blood flow [29,30], their contribution to brain maintenance remains uncertain. Notably, BPCs are closely linked with vascular cells throughout the brain, including those in the leptomeninges, and the glymphatic system’s perivascular tunnels extending into the brain. Therefore, when L-PGDS is released from BPCs, it may spread widely across the brain and CSF via the glymphatic system, potentially helping to regulate brain homeostasis.

Therefore, chemical compounds capable of increasing L-PGDS production from BPCs could help treat various brain diseases, including AD, CVD, and sleep disorders. In this study, we utilized compound libraries to identify chemical compounds capable of inducing L-PGDS production in BPCs.

## 2. Materials and Methods

### 2.1. Cell Culture

Commercially available mouse-derived brain pericytes (m-BPCs, #M1200, ScienCell Research Laboratories, Carlsbad, CA, USA) (3 × 10^4^ cells/cm^2^) were cultured in dishes according to the manufacturer’s protocol. At 24 h after incubation, the medium was replaced by fresh Dulbecco’s Modified Eagle’s Medium/Nutrient Mixture F-12 (DMEM/F12, Thermo Fisher Scientific, Waltham, MA, USA). Then, m-BPCs were treated with Tocriscreen FDA-Approved Drugs Library (100 µM, #7200, Tocris, Abingdon, Oxfordshire, UK) or dimethyl sulfoxide (DMSO) as vehicle control, followed by additional incubation for 3 days. Then, the collected cells were subjected to reverse transcription quantitative polymerase chain reaction (RT-qPCR) to measure L-PGDS expression.

In some experiments, m-BPCs were treated with different MTZ doses (0, 0.5, 5, 50, 100 µM, Tocris) or DMSO (control) and incubated for 3 days. After capturing images by a microscope (Olympus, Tokyo, Japan), the collected cells underwent RT-qPCR analysis.

In another set of experiments, Mianserin hydrochloride (MH, Tokyo Chemical Industry, Tokyo, Japan) was identified as a structurally similar compound to MTZ through a similarity search on the website of Tokyo Chemical Industry (TBI) (https://www.tcichemicals.com/JP/ja/structure-search (accessed on 19 November 2025)). M-BPCs were treated with MH (100 µM) and incubated for 3 days. Then, the collected cells underwent RT-qPCR analysis.

In addition, human-derived brain pericytes (h-BPCs) (#1200, ScienCell Research Laboratories) (3 × 10^4^ cells/cm^2^) were cultured in dishes according to the manufacturer’s protocol. At 24 h after incubation, the medium was replaced by fresh DMEM/F12 supplemented with 2% fetal bovine serum (FBS, Thermo Fisher Scientific) and different MTZ doses (0, 0.5, 5, 50, 100 µM) or DMSO (control) for 3 days. After capturing images using a microscope (Olympus), the collected cells and culture supernatant were subjected to RT-qPCR and Western blot (WB) analysis, respectively.

### 2.2. Reverse Transcription Quantitative Polymerase Chain Reaction

Total RNA was isolated from culture cells using a CellAmp Direct RNA Prep Kit (Takara Bio Inc., Shiga, Japan) as previously described [17]. In brief, complementary DNA was synthesized using PrimeScript RT Master Mix (Takara Bio), and qPCR was performed using TB Green Fast qPCR Mix (Takara Bio). The primer sequences used in this study were as follows: mouse L-PGDS: 5′-AGTGGTGGAGGCCAACTATG-3′ (forward) and 5′-TCTCCTTCAGCTCGTCCTTC-3′ (reverse); mouse β-actin: 5′-CGCGAGCACAGCTTCTTTG-3′ (forward) and 5′-CGTCATCCATGGCGAACTGG-3′ (reverse); human L-PGDS: 5′-GGCGTTGTCCATGTGCAAG-3′ (forward) and 5′-GGACTCCGGTAGCTGTAGGA-3′ (reverse); and human β-actin: 5′-CCTGGCACCCAGCACAAT-3′ (forward) and 5′-GCCGATCCACACGGAGTACT-3′ (reverse). The thermal cycling conditions were as follows: denaturation for one cycle at 94 °C for 30 s, followed by 40 cycles at 94 °C for 5 s and 60 °C for 10 s. The amplification efficiencies ranged from approximately 90% to 110% (mouse L-PGDS; 101.8%, mouse β-actin; 102.7%, human L-PGDS; 98.4%, human β-actin; 89.6%). Relative mRNA expression levels were determined based on the ∆∆CT method. The expression level was normalized to that of β-actin and the normalized expression level used for statistical analysis.

### 2.3. Immunohistochemistry

M-BPCs were fixed with 4% paraformaldehyde and subjected to immunohistochemis try using primary antibodies against NG2 (1:250, rabbit, EMD Millipore Corporation, Temecula, CA, USA) and αSMA (1:100, mouse, EMD Millipore), followed by Alexa Fluor 555-conjugated secondary antibodies (1:500, Abcam, Cambridge, UK). Cell nuclei were stained with DAPI (1:500, Kirkegaard & Perry Laboratories, Inc., Gaithersburg, MD, USA), and images were captured using a confocal laser scanning microscope (LSM780; Carl Zeiss AG, Oberkochen, Germany) as previously described [17,27,31,32].

### 2.4. Western Blot Analysis

To examine L-PGDS protein expression, m-BPCs (3 × 10^4^ cells/cm^2^) were inoculated into dishes following the manufacturer’s protocol. After 24 h, the medium was replaced by fresh DMEM/F12. Then, m-BPCs were treated with different MTZ doses (0, 0.5, 5, 50, 100 µM) or DMSO (control) for 3 days. In another set of experiments, m-BPCs (3 × 10^4^ cells/cm^2^) were treated with DMSO (control) or MTZ (100 µM), and incubated for 1, 3, or 5 days.

Next, the culture supernatant was subjected to WB analysis as previously reported [17]. In brief, the collected supernatant (10 µL/lane) was subjected to sodium dodecyl sulfate-polyacrylamide gel electrophoresis. Then, separated proteins were transferred onto polyvinylidene difluoride membranes (Bio-Rad, Hercules, CA, USA), and the membranes were incubated with primary antibodies against L-PGDS (1:1000, mouse; Santa Cruz Biotechnology, Dallas, TX, USA). Thereafter, membranes were incubated with horseradish peroxidase-labeled secondary antibodies (1:1000, mouse; Cell Signaling Technology, Danvers, MA, USA). Protein bands were visualized using an enhanced chemiluminescence reagent (Chemi-Lumi One, Nacalai Tesque, Kyoto, Japan). L-PGDS expression was analyzed using ImageJ 1.54 software.

### 2.5. Microarray Analysis

Using the RNeasy Micro Kit (Qiagen, Hilden, Germany), total RNA was isolated from m-BPCs treated with MTZ (100 µM) or DMSO (control) for 3 days as previously reported [17,27]. Then, RNA samples (*n* = 1, for each group) underwent microarray analysis; the results were analyzed using the Affymetrix Transcriptome Analysis console as previously described [17,27]. Pathway analysis was conducted using WikiPathways as described [31,32]. Gene ontology (GO) analysis was performed using the Shiny GO tool [33].

### 2.6. Statistical Analysis

Data are displayed as mean ± SD. Normality was first assessed. For the comparisons between two groups, all data satisfied normality, but displayed heterogeneous variance using the *F* test. Therefore, Welch’s *t*-test was used for comparisons between two groups. Comparisons between multiple groups were performed using Bonferroni post hoc tests when the results of one-way analysis of variance were significant. *p*-values < 0.05 were considered statistically significant.

## 3. Results

### 3.1. MTZ Has a Strong Ability to Increase L-PGDS Levels in BPCs

**Figure 1:**
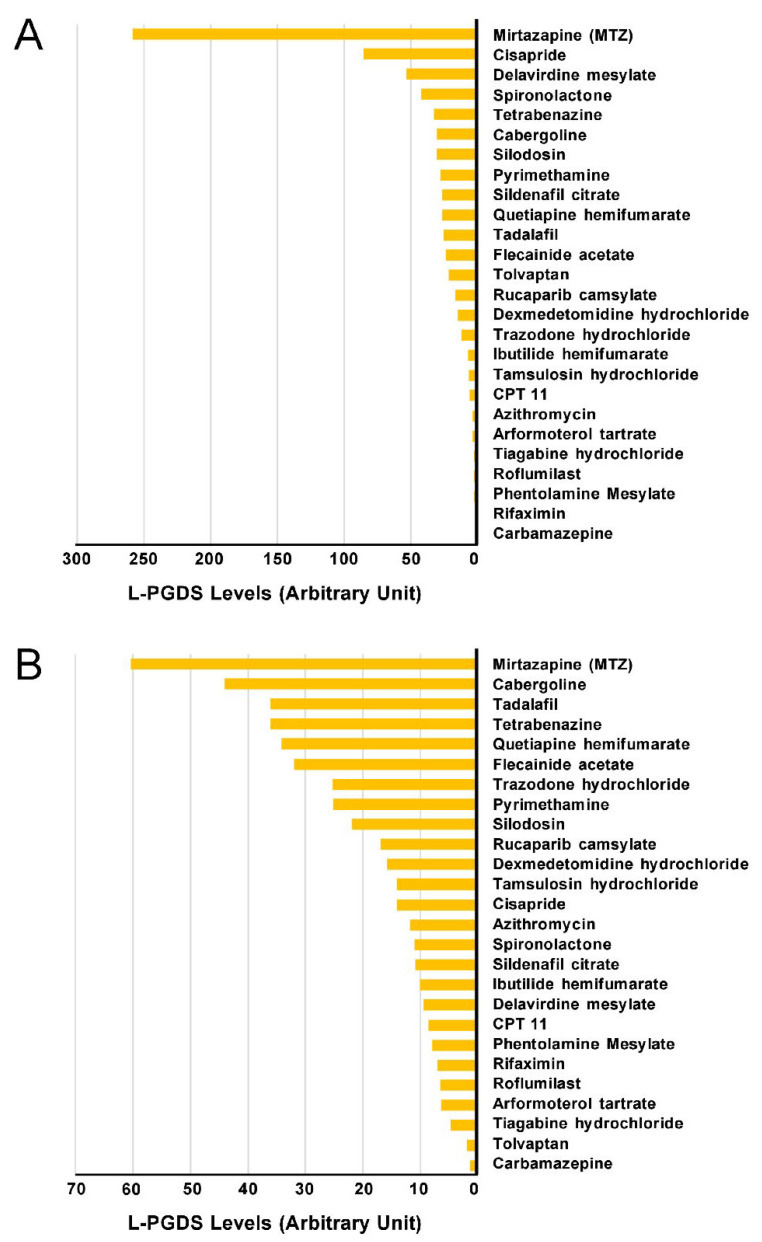
L-PGDS levels in m-BPCs after a treatment with 26 selected chemical compounds in the first (**A**) and second rounds (**B**) of experiments. MTZ was identified as the component with the greatest ability to increase L-PGDS expression in m-BPCs. Abbreviations: L-PGDS, lipocalin-type prostaglandin D synthase; m-BPCs, mouse-derived brain pericytes; and MTZ, mirtazapine.

Using 158 chemicals in FDA-approved drug libraries related to various research areas, including “Neuroscience,” “Cardiovascular,” “Stem Cells,” “Cancer,” “Endocrinology,” “Immunology,” and “Other” (Supplementary Tables S1–S3), we first screened chemical compounds inducing L-PGDS production. To study this, m-BPCs were treated with chemical compounds at a dose of 100 µM. On day 3 after treatment, samples were collected, and L-PGDS levels were measured by qPCR analysis. Compared with cells treated with vehicle control (DMSO), chemical compounds resulting in >15-fold expression (total 26 chemical compounds) were selected in the first screening (red font, Supplementary Tables S1–S3). Then, m-BPCs were treated with these chemicals at 100 µM. Among these 26 chemical compounds, L-PGDS levels were highest in BPCs after mirtazapine (MTZ) treatment (Figure 1A), as we confirmed in a subsequent test (Figure 1B). These results indicate that MTZ has the strong ability to increase L-PGDS levels in BPCs among chemical compounds used in this study.

### 3.2. MTZ Dose-Dependently Increases L-PGDS Levels in BPCs

**Figure 2:**
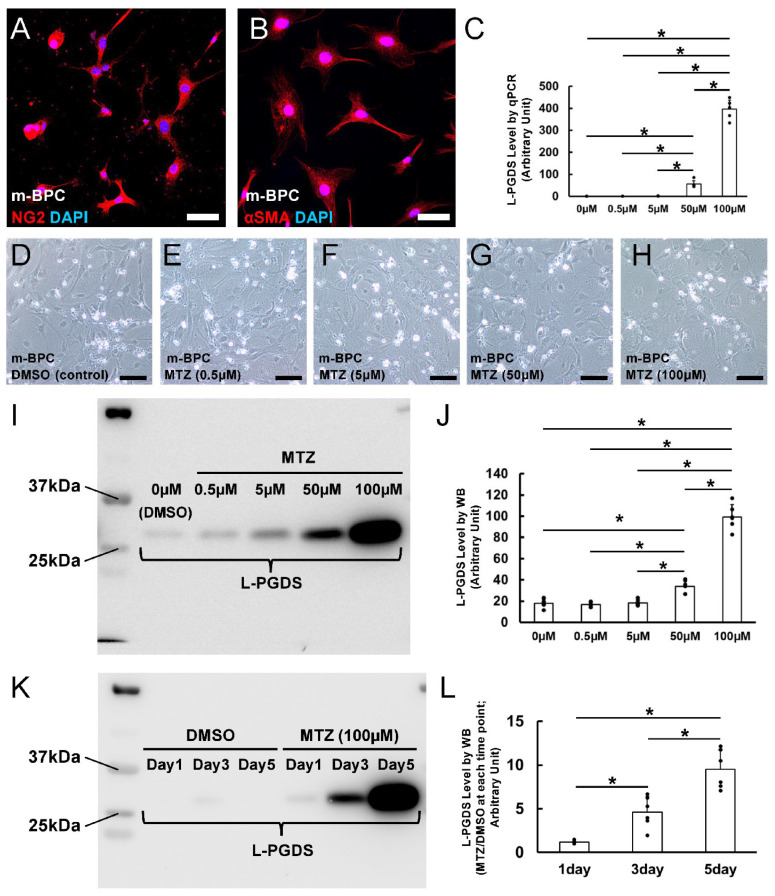
Immunohistochemistry for NG2 (NG2 [(**A**): red]; DAPI [(**A**): blue]) and αSMA (αSMA [(**B**): red]; and DAPI [(**B**): blue]) in m-BPCs. RT-qPCR analysis for L-PGDS levels in m-BPC (**C**) treated with DMSO (**D**) and 0.5 µM (**E**), 5 µM (**F**), 50 µM (**G**), and 100 µM (**H**) of MTZ over 3 days. WB analysis for L-PGDS levels in the culture supernatant of m-BPC treated with DMSO and 0.5 µM, 5 µM, 50 µM, and 100 µM of MTZ over 3 days (**I**,**J**). WB analysis for L-PGDS levels in the culture supernatant of m-BPC treated with DMSO and 100 µM of MTZ over 1, 3, or 5 days (**K**,**L**). Scale bars: 50 µm (**A**,**B**) and 100 µm (**D**–**H**). * *p* < 0.05 among groups (**C**,**J**,**L**). *n* = 6 (**C**,**J**,**L**) for each group. Abbreviations: αSMA, alpha-smooth muscle actin; DAPI, 4′,6-diamidino-2-phenylindole; DMSO, dimethyl sulfoxide; L-PGDS, lipocalin-type prostaglandin D synthase; m-BPCs, mouse-derived brain pericytes; MTZ, mirtazapine; NG2, neural/glial antigen 2; RT-qPCR, quantitative reverse transcription–polymerase chain reaction; and WB, Western blot. The original images are provided in the Supplementary Materials.

Next, BPCs were treated with various MTZ concentrations and L-PGDS production was investigated. To this end, m-BPCs expressing typical markers such as NG2 (Figure 2A) and αSMA (Figure 2B), were treated with DMSO (control) or 0.5 µM, 5 µM, 50 µM, and 100 µM of MTZ for 3 days, and L-PGDS levels were analyzed for each sample by qPCR (Figure 2C). Compared with DMSO treatment (Figure 2D), no morphological changes were observed in BPCs at 0.5 µM (Figure 2E), 5 µM (Figure 2F), 50 µM (Figure 2G), or 100 µM (Figure 2H) of MTZ. However, qPCR analysis showed that MTZ treatment significantly increased L-PGDS levels in a dose-dependent manner (Figure 2C).

Since L-PGDS is a secretory protein, we next measured L-PGDS levels in the culture supernatant. To study this, m-BPCs were treated with DMSO (control) or 0.5 µM, 5 µM, 50 µM, and 100 µM of MTZ for 3 days. WB analysis showed significantly higher L-PGDS levels with increasing MTZ doses (Figure 2I,J).

We next investigated L-PGDS protein levels at various time points after MTZ treatment. To study this, m-BPCs were treated with MTZ (100 µM) or DMSO (control) for 5 days. On days 1, 3, and 5, the culture supernatant was collected and subjected to WB analysis (Figure 2K). Normalized by DMSO control at the corresponding time point, L-PGDS levels were significantly higher at later time points (Figure 2L).

### 3.3. MTZ’s Chemical Structure May Promote L-PGDS Production in BPCs

MTZ is a noradrenergic and specific serotonergic antidepressant (NaSSA), promoting noradrenalin and serotonin release from neurons by regulating α2 and serotonin receptors [34]. However, MTZ treatment was not performed on neurons in this study. Thus, noradrenalin and/or serotonin do not appear directly related to the MTZ-derived L-PGDS.

To test the hypothesis, we next investigated L-PGDS levels in BPCs after treatment with serotonin–noradrenalin reuptake inhibitors (SNRI). As expected, compared with the DMSO-treated control, L-PGDS levels in BPCs were only 2.52-fold higher or were not changed after treatment with venlafaxine hydrochloride (Cat. No 2917) (Supplementary Table S1) or (S)-Duloxetine hydrochloride (Cat No 4798) (Supplementary Table S3), respectively. In addition, increased L-PGDS levels in BPCs were observed for various chemicals with targets different from MTZ (Supplementary Tables S1–S3). These results suggest that increased L-PGDS levels in BPCs are not likely due to the pharmacological effect related to NaSSA and/or SNRI.

**Figure 3:**
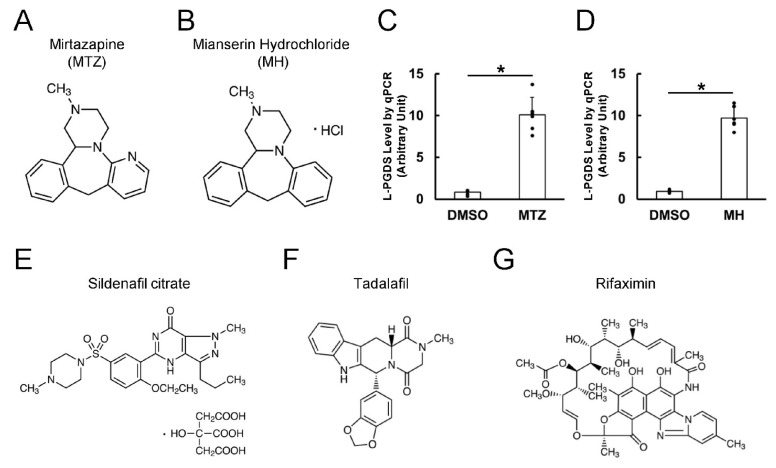
L-PGDS levels examined using MTZ (**A**) or MH (**B**), which have a similar chemical structure to MTZ (78% similarity). RT-qPCR analysis for L-PGDS levels in m-BPCs treated with DMSO and 100 µM MTZ over 3 days (**C**). RT-qPCR analysis for L-PGDS levels in m-BPCs treated with DMSO and 100 µM of MH for 3 days (**D**). Among the 26 selected chemical compounds in Figure 1, several chemicals, such as sildenafil citrate (**E**), tadalafil (**F**), and rifaximin (**G**), were identified as chemicals with a similar structure to MTZ (60% similarity). * *p* < 0.05 between groups (**C**,**D**). *n* = 6 (**C**,**D**) for each group. Abbreviations: DMSO, dimethyl sulfoxide; L-PGDS, lipocalin-type prostaglandin D synthase; m-BPCs, mouse-derived brain pericytes; MH, mianserin hydrochloride; MTZ, mirtazapine; and RT-qPCR, quantitative reverse transcription–polymerase chain reaction.

Next, we asked whether the chemical structure of MTZ (Figure 3A) may be responsible for promoting L-PGDS production in BPCs. To investigate this, m-BPCs was treated with MH (Figure 3B), whose chemical structure is very similar to MTZ (78% similarity). The results showed that, compared with the DMSO-treated control, there were significantly increased L-PGDS mRNA levels in BPCs treated with MH (Figure 3D) to a similar degree as that obtained with the same MTZ dose (Figure 3C).

We also observed that several chemical compounds with a similar structure to MTZ (60% of similarity), such as sildenafil citrate (Figure 3E), tadalafil (Figure 3F), and rifaximin (Figure 3G), were included in the 26 selected chemical compounds (Figure 1A,B).

### 3.4. Phenotypic Changes in Gene Expression Patterns of BPCs After MTZ Treatment

**Figure 4:**
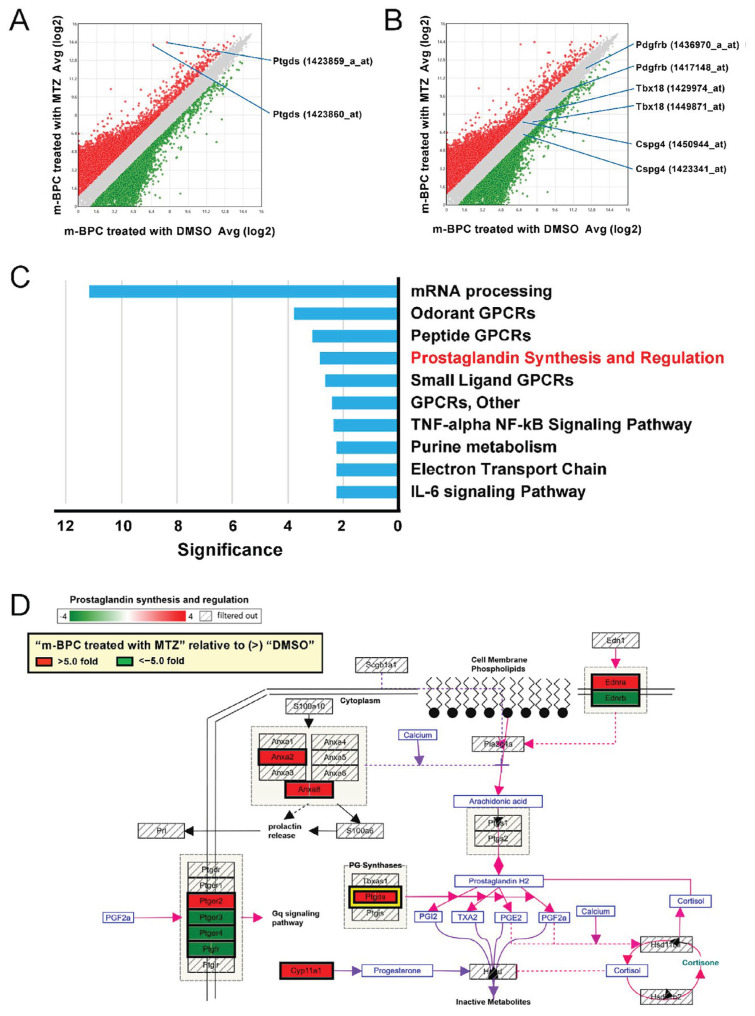
Scatter plot analysis by microarray showing the distribution of genes, with significantly higher (>2-fold, red plots) or lower (<−2-fold, green plots) expression in m-BPCs treated with MTZ compared with DMSO (**A**). Scatter plot analysis by microarray showing the distribution of genes related to pericytic markers, such as PDGFRβ, NG2 (Cspg4), and Tbx18 (**B**). Pathway analysis using WikiPathways by targeting genes significantly upregulated (>5-fold) or downregulated (<−5-fold) in m-BPCs after MTZ treatment compared to DMSO treatment (**C**). The results of the top ten categories displayed the presence of the term “Prostaglandin synthesis and regulation” (red font) (**C**), and L-PGDS (Ptgds) outlined in yellow font in this category (**D**). Abbreviations: DMSO, dimethyl sulfoxide; L-PGDS, lipocalin-type prostaglandin D synthase; m-BPCs, mouse-derived brain pericytes; MTZ, mirtazapine; NG2, neural/glial antigen 2; PDGFRβ, platelet-derived growth factor receptor beta; and Tbx18, T-box transcription factor 18.

To further investigate the gene expression patterns of BPCs after MTZ treatment, m-BPCs were treated with DMSO (control) or MTZ for 3 days. Then, collected samples were subjected to microarray analysis. Table 1 shows the top ten highly expressed genes in m-BPCs treated with MTZ compared with those treated with vehicle controls (DMSO). L-PGDS (*Ptdgs*) was listed as the top one and three highly expressed genes (Table 1). Scatter plot analysis showed genes with expression levels over two-fold higher (red) or lower (green) in m-BPCs treated with MTZ versus DMSO, confirming that MTZ significantly upregulated L-PGDS expression in BPCs (Figure 4A). In contrast, various pericytic markers, such as PDGFRβ, CSPG4, and Tbx18, did not significantly differ between groups (Figure 4B). These results suggest that MTZ has the strong ability to produce L-PGDS from BPCs while maintaining their basic traits. To further investigate the influence of MTZ treatment on BPCs, we next performed pathway analysis by targeting genes significantly upregulated (>5-fold) or downregulated (<−5-fold) in m-BPCs after MTZ treatment. The top ten categories included the term “Prostaglandin synthesis and regulation” (Figure 4C), comprising significantly upregulated (e.g., *Anxa2*, *Anxa8*, *Cyp11a1*, *Ednra*, *Ptgds*, and *Ptger2*, in red) and/or downregulated genes (e.g., *Ednrb*, *Ptger3*, *Ptger4*, and *Ptgfr*, in green) (Figure 4D). “*Ptgds*” is outlined by the yellow box (Figure 4D).

**Figure 5:**
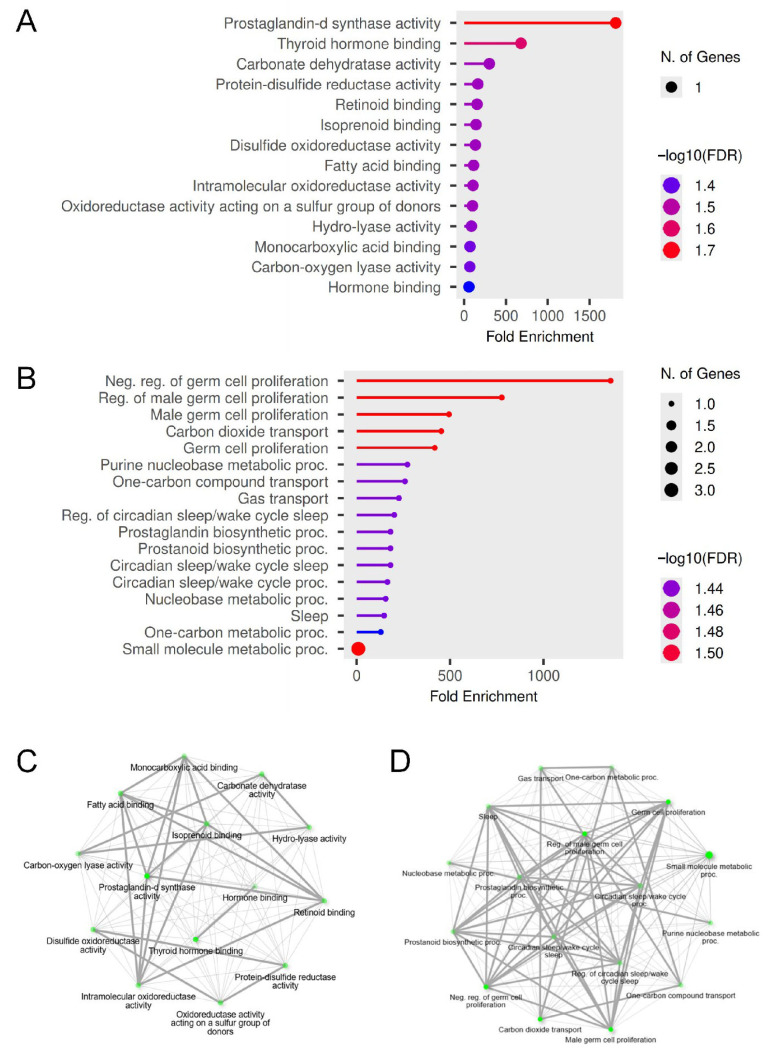
Gene ontology (GO) analysis by targeting genes highly upregulated (>50-fold) in m-BPCs after MTZ compared to DMSO treatment (**A**). The term “Prostaglandin-d synthase activity” was present by the “GO molecular function” analysis (**A**). Several terms “prostaglandin biosynthetic proc.,” “Prostanoid biosynthetic proc.,” “Circadian sleep/wake cycle sleep,” “Circadian sleep/wake cycle proc.,” and “Sleep” were present by “GO biological process” analysis (**B**). Related GO terms are connected by lines (**C**,**D**).

We further performed GO analysis by targeting highly upregulated genes (>50-fold) in m-BPCs after MTZ treatment compared to DMSO-treated controls. “GO molecular function” analysis included the term “Prostaglandin-d synthase activity” (Figure 5A). The associated GO terms were connected by a line (Figure 5C). “GO biological process,” analysis included the term “Prostaglandin biosynthetic proc.,” “Prostanoid biosynthetic proc.,” “Circadian sleep/wake cycle sleep,” “Circadian sleep/wake cycle proc.,” and “Sleep” (Figure 5B). The related GO terms showed that these terms were connected by a line (Figure 5D).

### 3.5. L-PGDS Production in Human BPCs After MTZ Treatment

**Figure 6:**
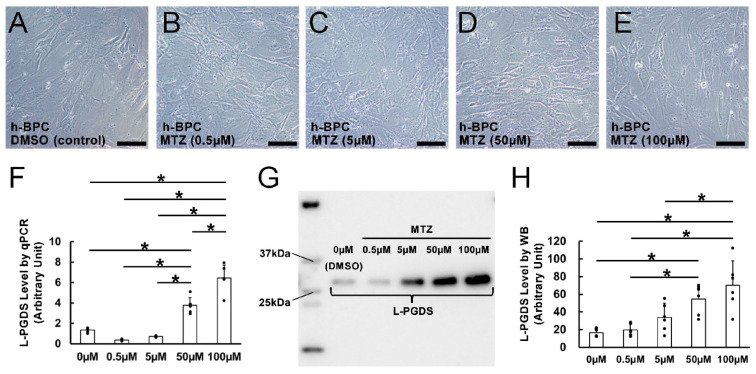
RT-qPCR analysis for L-PGDS levels in h-BPC treated with DMSO (**A**) and 0.5 µM (**B**), 5 µM (**C**), 50 µM (**D**), and 100 µM (**E**) of MTZ over 3 days (**F**). WB analysis for L-PGDS levels in the culture supernatant of h-BPC treated with DMSO and 0.5 µM, 5 µM, 50 µM, and 100 µM of MTZ over 3 days (**G**,**H**). Scale bars: 100 µm (**A**–**E**). * *p* < 0.05 among groups (**F**,**H**). *n* = 6 (**F**,**H**) for each group. Abbreviations: DMSO, dimethyl sulfoxide; h-BPCs, human-derived brain pericytes; L-PGDS, lipocalin-type prostaglandin D synthase; MTZ, mirtazapine; RT-qPCR, quantitative reverse transcription–polymerase chain reaction; and WB, Western blot.

Finally, we investigated whether h-BPCs can produce L-PGDS after MTZ treatment. To this end, h-BPC were treated with DMSO (control) (Figure 6A) and 0.5 µM (Figure 6B), 5 µM (Figure 6C), 50 µM (Figure 6D), and 100 µM (Figure 6E) of MTZ over 3 days. qPCR analysis showed that a higher MTZ dose significantly increased L-PGDS levels (Figure 6F).

Further, h-BPCs were treated with DMSO (control) or 0.5 µM, 5 µM, 50 µM, and 100 µM of MTZ for 3 days. Then, the supernatant was collected, and L-PGDS levels were measured. WB analysis showed significantly upregulated L-PGDS levels by higher MTZ doses (Figure 6G,H). These results indicate that MTZ has the activities to produce L-PGDS from h-BPC as well as m-BPC, suggesting that L-PGDS-producing ability after MTZ treatment is common among BPCs of different species.

### 3.6. Schematic Illustration of Possible Target Diseases by MTZ-Derived L-PGDS Production

**Figure 7:**
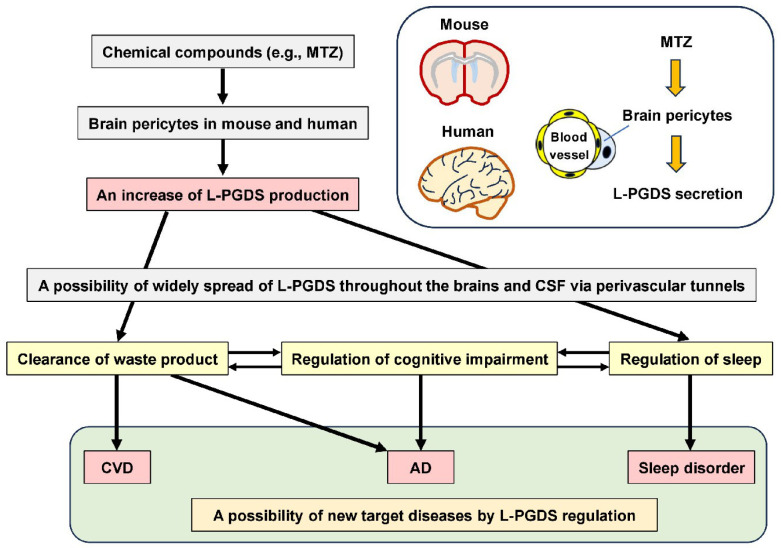
Schematic representation of the proposed association of L-PGDS produced by chemical compounds (e.g., MTZ) in BPCs and possible new target diseases, such as CVD, AD, and sleep disorders. Abbreviations: AD, Alzheimer’s disease; BPCs, brain pericytes; CSF, cerebrospinal fluid; CVD, cerebrovascular disease; L-PGDS, lipocalin-type prostaglandin D synthase; and MTZ, mirtazapine.

A schematic overview illustrating the proposed connections between MTZ-derived L-PGDS production in BPCs and potential new target diseases—such as AD, CVD, and sleep disorders—is provided (Figure 7), highlighting drug repositioning possibilities through L-PGDS regulation.

## 4. Discussion

Under physiological conditions, the brain maintains homeostasis partially through the clearance of waste products. However, the failure of the clearance function would cause the accumulation of waste products, such as Aβ and Tau, in the brains, possibly triggering the onset of various neurodegenerative diseases, including AD [2,3]. Although the precise mechanism by which waste product is removed from the brains remains unclear, recent studies have proposed that the brains have unique perivascular tunnels called the glymphatic system [5,6]. Further, accumulating studies indicate that the dysfunction of the glymphatic system is associated with the pathogenesis of cognitive impairments and sleep disorders [8]. Under pathological conditions, excessive waste product accumulates in the injured brains. For example, debris such as biliverdin and fragments of damaged myelin emerged after brain hemorrhage and ischemic stroke, and the accumulation of excessive waste product would prevent the repair and regeneration of brain tissues. Therefore, it is very important to regulate the clearance of waste products appropriately.

L-PGDS has a high affinity for low-molecular-weight hydrophobic compounds with an eight-stranded antiparallel β-barrel structure [35], and functions as a scavenger for Aβ [9,10] and biliverdin [11]. L-PGDS and/or the L-PGDS-PGD2-DP1 axis are also associated with the pathogenesis of ischemic stroke [14,15,16,36,37]. The exact function of the L-PGDS-PGD2-DP1 axis in ischemic stroke is unclear, but recent findings show that PGD2 induces MGs/MΦs to switch to CD36+ scavenger types, activating the L-PGDS-PGD2-CD36 pathway post-stroke [1,17]. CD36+ MGs/MΦs are capable of clearing debris in ischemic regions [17]. Thus, L-PGDS-inducible chemical compounds could promote the clearance of debris within injured brain areas, thereby accelerating brain repair after ischemic stroke.

In this study, using various FDA-approved drug libraries, we investigated chemical compounds capable of inducing L-PGDS production in BPCs. L-PGDS levels were elevated in BPCs treated with chemicals related to various research areas, including “Neuroscience,” “Cardiovascular,” “Stem Cells,” “Cancer,” “Endocrinology,” “Immunology,” and “Other.”. In particular, MTZ had the strongest ability to induce L-PGDS production in BPCs, suggesting that MTZ administration can trigger L-PGDS-derived biological effects in the brain. However, the orders of L-PGDS levels in BPCs differed between the first and second rounds of experiments for some chemical compounds. Although we do not know exactly how this may have influenced the outcomes, several conditions (e.g., cell conditions such as cell viability and impurity of the culture supernatant) may have affected the results, leading to a change in the order of some chemical compounds, particularly when the L-PGDS values were similar. However, for the first and second rounds of experiments, MTZ was the highest and carbamazepine was the lowest. Therefore, even if these factors may have influenced the experimental results, it remains likely that MTZ had the highest activity to induce L-PGDS among the listed chemical compounds.

In this study, SNRIs, such as venlafaxine hydrochloride or (S)-Duloxetine hydrochloride, with a similar effect to NaSSAs, had barely any effect on L-PGDS levels in BPCs. Increased L-PGDS levels in BPCs were also noted for chemicals with targeting sites different from MTZ. These results indicate that the increase in L-PGDS observed in BPCs treated with MTZ does not appear to be related to its pharmacological effects by NaSSAs. Mirtazapine possesses a tetracyclic piperazino-azepine structure with the chemical formula C17H19N3 and originally developed from MH. In this study, BPCs treated with MH also demonstrated a significant increase in L-PGDS production, comparable to the levels observed with MTZ treatment. These results suggest that the chemical structure of MTZ may be responsible for activating L-PGDS production in BPCs, although further studies are needed to identify the chemical structures responsible for L-PGDS production in BPCs.

The L-PGDS-PGD2-DP1 axis is involved in regulating sleep [24]. In addition, a recent study showed that L-PGDS levels are elevated in patients with hypersomnia [25]. Therefore, chemical compounds which increase L-PGDS levels would be useful for patients with insomnia in the form of sleeping pills, whereas chemical compounds which decrease L-PGDS levels would be useful for patients with hypersomnia (e.g., narcolepsy). In the present study, we found that MTZ has a strong ability to produce and release L-PGDS from BPCs. It remains unclear whether MTZ causes sleepiness by increasing the L-PGDS levels in humans. However, package inserts of MTZ described that MTZ accompanies excessive sleep at high frequency (50%). Although it has been primarily considered to be attributed to the MTZ-related effect regarding blocking serotonin 2A and histaminergic H1 receptors [38], evidence that excessive sleep is highly observed in patients after MTZ treatment suggests the possibility of additional mechanisms, such as increased L-PGDS levels in the brain. Because patients with depression frequently accompany sleep disorders, MTZ may be useful not only as an antidepressant drug but also as sleeping pills with a novel mechanism targeting the L-PGDS-PGD2-DP1 axis. Although further studies are needed to clarify this view point, accumulating evidence shows that MTZ improved the quality of sleep and prolonged the duration of sleep [39,40,41]. In addition, a recent study demonstrated the usefulness of MTZ as sleeping pills for patients with chronic insomnia [38].

Pathway analysis showed, in addition to “Prostaglandin synthesis and regulation,” several upregulated inflammatory-related pathways, such as the “TNF-alpha NF-kΒ signaling pathway” and “IL-6 signaling pathway,” in BPCs after MTZ treatment. These results suggest that MTZ has an effect on inflammation. Accordingly, accumulating evidence shows that MTZ modulates neuroinflammation [42], including regulating NF-κB/TLR4 signaling [43]. Moreover, MTZ has various bioactive effects, including anti-inflammatory, antioxidative, and anti-apoptotic effects [34,44,45]. Therefore, MTZ may have the potential to treat other diseases, such as diabetes, or liver or kidney disease [34].

This study found that BPCs produce L-PGDS following MTZ treatment. Pericytes are a heterogeneous group of cells expressing markers like PDGFRβ, NG2, αSMA, and Tbx18 [46,47,48]; thus, their responses to chemicals may vary by subtype. It remains unknown which BPC subsets yield the most L-PGDS, or if chemical specificity exists beyond MTZ. Future research should address these questions.

Previous studies showed that L-PGDS was expressed by cells constructing the NVU, including BPCs [17], astrocytes (ACs) [49], and endothelial cells (ECs) [27,28,50,51]. Although whether ACs and ECs can produce L-PGDS after MTZ treatment remains unclear, our preliminary results showed a lower increase in L-PGDS after MTZ treatment in ACs than in BPCs. In addition, MTZ treatment did not affect L-PGDS levels in ECs. Among the cells comprising the neurovascular unit (NVU), BPCs are likely significant contributors to L-PGDS production. Although the mechanisms through which the L-PGDS produced by BPCs is distributed throughout the brain remain uncertain, this study demonstrated that MTZ enhances L-PGDS secretion from BPCs at both mRNA and protein levels. Therefore, L-PGDS protein secreted in response to MTZ may be dispersed broadly within the brain and CSF via specific pathways associated with BPCs, such as perivascular tunnels (glymphatic system) and the leptomeninges.

Although the precise roles of BPCs remain unclear, it has been documented that the dysfunction of BPCs is closely associated with the pathogenesis of cognitive impairment, such as AD [52]. In addition, PC transplantation reduced Aβ deposition in a mouse model of AD [53]. Insufficient L-PGDS production by dysfunctional BPCs may result in increased Aβ deposition. Although the impact of MTZ on BPCs in pathological brains is still unclear, previous studies have shown that BPCs resist injury, such as ischemic stroke [27,54], and can produce L-PGDS even under pathological conditions [17]. Thus, chemicals promoting L-PGDS production from BPCs may be beneficial for pathological brains. However, additional studies are needed to determine whether MTZ can produce L-PGDS in BPCs in vivo using an animal model of AD, thereby having a useful effect in the brain through the L-PGDS-associated mechanism (e.g., scavenging waste products). This study found MTZ-induced L-PGDS production in m-BPCs and h-BPCs, suggesting that MTZ could increase L-PGDS levels in human brains via h-BPC secretion. In this study, L-PGDS was significantly induced in BPCs (both m-BPCs and h-BPCs) treated with ≥50 µM MTZ. However, the package inserts for MTZ state that the Cmax is approximately 85 ng/mL when adult humans receive 30 mg MTZ. Therefore, the uptake of a higher dose of MTZ than the regular dose may be clinically needed to produce L-PGDS in BPCs in humans. The L-PGDS-producing effect in the human brain therefore requires further investigation.

## 5. Conclusions

In summary, we demonstrated for the first time that chemical compounds have the ability to produce L-PGDS in BPCs. In addition, we showed that MTZ can induce BPCs to secrete L-PGDS, highlighting the importance of BPCs as not only sources of maintaining brain homeostasis but also as providing the possibility of a new target for repairing brain diseases, including neurovascular and neurodegenerative diseases, by assisting the glymphatic system. There are many issues to be clarified. For example, whether MTZ can promote L-PGDS production in vivo or whether activated L-PGDS by MTZ triggers downstream cascades, such as the PGD2-DP1 or PGD2-CD36 axis, remains unclear. To detect L-PGDS-related signaling pathways, receptors, or mediators in greater detail, the relationships between cell–cell interactions within BPCs as well as between BPCs and other cell types should be clarified in future studies (e.g., in vivo animal models, in vitro co-culture experiments). In addition, the maximum dose of MTZ that can produce L-PGDS in human brains without critical side effects should be elucidated in future studies. However, the present study has demonstrated for the first time that chemical compounds, such as MTZ, have a strong ability to produce L-PGDS in BPCs at both the gene and protein levels. Thus, MTZ may have the potential to be applied as drug repositioning that targets various brain diseases, including AD, CVD, and sleep disorders, by regulating L-PGDS and/or L-PGDS-related cascades, although these issues require further investigation.

## Figures and Tables

**Table 1:** List of top ten genes highly expressed in m-BPCs treated with MTZ relative to m-BPCs treated with DMSO (control).

Gene Symbol	Gene Name	ID	Fold Change
*Ptgds*	prostaglandin D2 synthase (brain)	1423860_at	202.14
*Car14*	carbonic anhydrase 14	1450725_s_at	179.35
*Ptgds*	prostaglandin D2 synthase (brain)	1423859_a_at	100.11
*Ttr*	transthyretin	1454608_x_at	84.4
*Txnl1*	thioredoxin-like 1	1437906_x_at	54.42
*Galnt15*	UDP-N-acetyl-alpha-D-galactosamine:polypeptide N-acetylgalactosaminyltransferase 15	1429235_at	45.92
*Cldn2*	claudin 2	1417231_at	41.84
*Ntm*	neurotrimin	1458492_x_at	41.74
*Myom2*	myomesin 2	1450917_at	36.57
*Ablim3*	actin binding LIM protein family, member 3	1434013_at	36.35

## Data Availability

The data supporting this article will be shared by the corresponding author upon reasonable request.

## Supplementary Materials

The following supporting information can be downloaded at https://www.mdpi.com/article/10.3390/biom16070945/s1. Table S1: L-PGDS expression levels of m-BPCs after treatment with chemical compounds (No.1); Table S2: L-PGDS expression levels of m-BPCs after treatment with chemical compounds (No.2); Table S3: L-PGDS expression levels of m-BPCs after treatment with chemical compounds (No.3); Original Western Blot images.
