# Supplementary material for: Mirtazapine Induces Lipocalin-Type Prostaglandin D Synthase Expression in Brain Pericytes
Source: Biomolecules. 2026 Jun 24;16(7):945. doi: 10.3390/biom16070945 (PMC13407077; doi:10.3390/biom16070945)
Supplement: Supplementary file 1 [file biomolecules-16-00945-s001.zip › biomolecules-4392781-for final supplementary.pdf]

Supplementary Table 1

L-PGDS expression levels of m-BPCs after treatment with chemical compounds (No.1)

Cat. No	Compound name	L-PGDS level	Research area	Target Class	Primary Target
0691	Dipyridamole	0.982443902	Neuroscience	Transporters	Nucleoside Transporters
0713	Cyclothiazide	3.438772205	Neuroscience	Ion Channels	AMPA Receptors
0869	Felbamate	4.192217111	Neuroscience	Ion Channels	NMDA Receptors
0902	Cimetidine	2.635072127	Neuroscience	7-TM Receptors	Histamine H2 Receptors
0909	Tropicamide	3.805146306	Neuroscience	7-TM Receptors	M4 Receptors
0927	Fluoxetine hydrochloride	ND	Stem Cells	Transporters	5-HT Transporters
0937	Pimozide	ND	Neuroscience	7-TM Receptors	Non-selective Dopamine
0964	Diazoxide	5.246469631	Neuroscience	Ion Channels	AMPA Receptors
0999	Tamoxifen citrate	ND	Cancer	Nuclear Receptors	Estrogen and Related Receptors
1126	Dexamethasone	1.769758098	Stem Cells	Nuclear Receptors	Glucocorticoid Receptor
1226	Etoposide	2.04407503	Cancer	Enzymes	DNA Topoisomerase
1328	Flumazenil	4.847556102	Neuroscience	Ion Channels	GABAA Receptors
1453	Clemastine fumarate	ND	Neuroscience	7-TM Receptors	Histamine H1 Receptors
1470	Flecainide acetate	33.8770914	Neuroscience	Ion Channels	Voltage-gated Sodium Channels
1479	Mifepristone	ND	Endocrinology	Nuclear Receptors	Progesterone Receptor
1505	Mycophenolic acid	8.154690365	Cancer	Enzymes	Other Dehydrogenases
1530	Lovastatin	ND	Endocrinology	Enzymes	HMG-CoA Reductase
1637	Argatroban	8.342157177	Endocrinology	Enzymes	Other Proteases
1692	Cilostazol	0.505415809	Neuroscience	Enzymes	Phosphodiesterases
1695	Cisapride	96.65606042	Neuroscience	7-TM Receptors	5-HT4 Receptors
1706	Acetaminophen	14.76240242	Neuroscience	Enzymes	Cyclooxygenase
1944	Loratidine	0.308478182	Neuroscience	7-TM Receptors	Histamine H1 Receptors
1965	Simvastatin	ND	Endocrinology	Enzymes	HMG-CoA Reductase
2004	Isradipine	ND	Neuroscience	Ion Channels	Cav1.x Channels
2007	Fluticasone propionate	0.002719545	Endocrinology	Nuclear Receptors	Glucocorticoid Receptor
2018	Mirtazapine	48.85658268	Neuroscience	7-TM Receptors	Non-selective 5-HT2
2175	Tetrabenazine	34.69342156	Neuroscience	Transporters	Vesicular Monoamine Transporters
2252	Doxorubicin hydrochloride	7.0501554	Cancer	Enzymes	DNA Topoisomerase
2280	Raloxifene hydrochloride	6.767289708	Cancer	Nuclear Receptors	Estrogen and Related Receptors
2429	Fexofenadine hydrochloride	3.976575249	Neuroscience	7-TM Receptors	Histamine H1 Receptors
2513	Acyclovir	9.128098771	Cancer	Enzymes	RNA/DNA Polymerase
2571	Amlodipine besylate	ND	Neuroscience	Ion Channels	Cav1.x Channels
2578	Benazepril hydrochloride	0.999777059	Cardiovascular	Enzymes	Angiotensin-Converting Enzyme
2596	Mexiletine hydrochloride	9.106621626	Neuroscience	Ion Channels	Voltage-gated Sodium Channels
2600	Clofarabine	1.973135913	Cancer	Enzymes	Other Kinases
2624	Decitabine	5.423458175	Cancer	Enzymes	DNA Methyltransferases
2625	Zonisamide	2.423258866	Neuroscience	Ion Channels	Other Channel Modulators
2664	Cabergoline	104.5467539	Neuroscience	7-TM Receptors	Non-selective Dopamine
2671	Budesonide	0.248386345	Endocrinology	Nuclear Receptors	Glucocorticoid Receptor
2673	Acarbose	7.009104683	Cancer	Enzymes	Glycosylases
2682	Sodium 4-Phenylbutyrate	9.837245988	Cancer	Enzymes	Non-selective HDACs
2685	Carvedilol	ND	Cardiovascular	7-TM Receptors	Non-selective Adrenergic Beta Receptors
2688	CPT 11	22.16817554	Cancer	Enzymes	DNA Topoisomerase
2706	Temozolomide	3.934407537	Cancer	Cell biology	Apoptosis Inducers
2749	Dexmedetomidine hydrochloride	17.92836339	Cardiovascular	7-TM Receptors	Adrenergic Alpha-2 Receptors
2796	(S)-(+)-Ibuprofen	2.800665329	Neuroscience	Enzymes	Cyclooxygenase
2815	Valproic acid, sodium salt	1.770108575	Stem Cells	Enzymes	Non-selective HDACs
2839	Levetiracetam	2.192743293	Cancer	Cell biology	Translocation, Exocytosis & Endocytosis
2852	Adapalene	ND	Endocrinology	Nuclear Receptors	Retinoic Acid Receptors
2864	Metformin hydrochloride	2.786183182	Stem Cells	Enzymes	AMPK
2917	Venlafaxine hydrochloride	2.526317682	Neuroscience	Transporters	5-HT Transporters
2960	Felodipine	ND	Neuroscience	Ion Channels	Cav1.x Channels

Supplementary Tabel 2

L-PGDS expression levels of m-BPCs after treatment with chemical compounds (No.2)

2964	Doxazosin mesylate	ND	Cardiovascular	7-TM Receptors	Adrenergic Alpha-1 Receptors
2968	Spironolactone	22.52890123	Cancer	Nuclear Receptors	Mineralocorticoid Receptor
3000	Iressa	ND	Cancer	Enzyme-Linked Receptors	EGFR
3016	Miconazole nitrate	ND	Other	Other Pharmacology	Miscellaneous Compounds
3050	Tamsulosin hydrochloride	34.29723538	Cardiovascular	7-TM Receptors	Adrenergic Alpha-1 Receptors
3108	Bumetanide	1.76041386	Cardiovascular	Transporters	Na+/K+/Cl- Symporter
3109	Furosemide	2.623365779	Cardiovascular	Transporters	Na+/K+/Cl- Symporter
3118	Ranolazine dihydrochloride	10.90551846	Cancer	Enzymes	Other Dehydrogenases
3256	Metoprolol tartrate	2.755213846	Cardiovascular	7-TM Receptors	Adrenergic Beta-1 Receptors
3259	Gemcitabine hydrochloride	1.307560516	Cancer	Cell biology	DNA, RNA and Protein Synthesis
3292	Metyrapone	4.778651736	Cancer	Enzymes	Hydroxylases
3308	Zileuton	13.94710108	Cancer	Enzymes	Lipoxygenase
3309	Fluvastatin sodium	1.451245628	Endocrinology	Enzymes	HMG-CoA Reductase
3351	Selexipag	ND	Endocrinology	7-TM Receptors	Prostanoid Receptors
3495	Fludarabine	7.089659427	Cancer	Cell biology	DNA, RNA and Protein Synthesis
3517	Nebivolol hydrochloride	ND	Cardiovascular	7-TM Receptors	Adrenergic Beta-1 Receptors
3586	Sumatriptan succinate	1.360841854	Neuroscience	7-TM Receptors	5-HT1D Receptors
3609	Tizanidine hydrochloride	2.766826816	Cardiovascular	7-TM Receptors	Adrenergic Alpha-2 Receptors
3620	Topiramate	2.610832021	Neuroscience	Ion Channels	Kainate Receptors
3737	Asenapine maleate	0.889866986	Neuroscience	7-TM Receptors	Non-selective 5-HT
3754	Varenicline tartrate	1.55430978	Neuroscience	Ion Channels	Nicotinic (a4b2) Receptors
3756	Maraviroc	2.537155347	Immunology	7-TM Receptors	Chemokine CC Receptors
3757	Dofetilide	3.917665143	Neuroscience	Ion Channels	Voltage-Gated Potassium Channels
3759	Exemestane	0.500398449	Cancer	Enzymes	Cytochrome P450
3765	Linezolid	6.75935484	Other	Cell Biology	DNA, RNA and Protein Synthesis
3768	Sunitinib malate	ND	Cancer	Enzyme-Linked Receptors	VEGFR
3771	Azithromycin	24.86760516	Other	Cell biology	DNA, RNA and Protein Synthesis
3784	Sildenafil citrate	79.69727246	Neuroscience	Enzymes	Phosphodiesterases
3798	Losartan potassium	1.470299515	Cardiovascular	7-TM Receptors	Angiotensin AT1 Receptors
3805	Repaglinide	0.560264736	Cardiovascular	Ion Channels	Inward rectifier Potassium Channels
3852	Tranylcypromine hydrochloride	7.501743966	Stem Cells	Enzymes	Histone Demethylases
3863	Trovafloxacin mesylate	0.935079178	Other	Cell biology	DNA, RNA and Protein Synthesis
3896	Rotigotine hydrochloride	1.483454923	Neuroscience	7-TM Receptors	D2 Receptors
3908	Ibutilide hemifumarate	61.99683091	Neuroscience	Ion Channels	Other Channel Modulators
3918	Pyrimethamine	156.4485536	Immunology	Transporters	Multidrug Transporters
4056	Docetaxel	0.030452675	Cancer	Cell biology	Microtubules
4092	Aspirin	1.751053096	Neuroscience	Enzymes	Cyclooxygenase
4093	Hydrocortisone	3.855120514	Endocrinology	Nuclear Receptors	Glucocorticoid Receptor
4094	Flutamide	0.246861979	Cancer	Nuclear Receptors	Androgen Receptor
4096	Clotrimazole	2.072931081	Cancer	Enzymes	Cytochrome P450
4098	Carbamazepine	15.4095909	Neuroscience	Ion Channels	Voltage-gated Sodium Channels
4099	Azathioprine	2.40036203	Immunology	Cell Biology	DNA, RNA and Protein Synthesis
4102	Mycophenolate mofetil	5.175844512	Immunology	Enzymes	Other Dehydrogenases
4107	Probenecid	5.165924635	Cancer	Transporters	Multidrug Transporters
4117	Bepridil hydrochloride	ND	Neuroscience	Ion Channels	Non-selective Cav Channels
4124	Pioglitazone hydrochloride	2.908076017	Endocrinology	Nuclear Receptors	PPARgamma Receptors
4148	Abacavir hemisulfate	9.140882291	Cancer	Enzymes	RNA/DNA Polymerase
4149	Delavirdine mesylate	55.68883215	Other	Enzymes	RNA/DNA Polymerase
4216	Valsartan	4.478893963	Cardiovascular	7-TM Receptors	Angiotensin AT1 Receptors
4231	Nateglinide	3.818309331	Cardiovascular	Ion Channels	Inward rectifier Potassium Channels
4241	Olopatadine hydrochloride	4.19110912	Neuroscience	7-TM Receptors	Histamine H1 Receptors
4245	Meclizine dihydrochloride	ND	Neuroscience	Nuclear Receptors	Pregnane X Receptor
4256	Tiagabine hydrochloride	31.0446407	Neuroscience	Transporters	GABA Transporters

Supplementary Table 3

L-PGDS expression levels of m-BPCs after treatment with chemical compounds (No.3)

4308	Rasagiline mesylate	4.423182237	Neuroscience	Enzymes	Monoamine Oxidase
4418	Saquinavir mesylate	ND	Endocrinology	Enzymes	Other Proteases
4440	Rivastigmine tartrate	5.128055952	Neuroscience	Enzymes	Cholinesterases
4460	Trifluorothymidine	12.95824644	Neuroscience	Enzymes	Thymidylate Synthetase
4501	Ribavirin	5.324901859	Cancer	Enzymes	Protein-synthesizing GTPases
4553	Azilsartan	7.318572317	Cardiovascular	7-TM Receptors	Angiotensin AT1 Receptors
4616	Olmesartan	5.935112108	Cardiovascular	7-TM Receptors	Angiotensin AT1 Receptors
4652	SAHA	ND	Cancer	Enzymes	Non-selective HDACs
4657	Zolmitriptan	4.623702346	Neuroscience	7-TM Receptors	5-HT1D Receptors
4712	Salmeterol xinafoate	ND	Cardiovascular	7-TM Receptors	Adrenergic Beta-2 Receptors
4735	Quetiapine hemifumarate	105.2332321	Neuroscience	7-TM Receptors	Non-selective 5-HT2
4791	Candesartan	11.61282526	Cardiovascular	7-TM Receptors	Angiotensin AT1 Receptors
4798	(S)-Duloxetine hydrochloride	ND	Neuroscience	Transporters	5-HT Transporters
4799	Capecitabine	2.945612858	Cancer	Cell Biology	DNA, RNA and Protein Synthesis
4819	Methylprednisolone	3.801597201	Endocrinology	Nuclear Receptors	Glucocorticoid Receptor
4833	Levonorgestrel	5.061375163	Endocrinology	Nuclear Receptors	Progesterone Receptor
4857	Amlexanox	4.609555141	Immunology	Enzymes	TANK binding kinase (TBK)
4942	Pitavastatin calcium	0.080670967	Endocrinology	Enzymes	HMG-CoA Reductase
4985	Miglitol	7.073914058	Cancer	Enzymes	Glycosylases
4990	Stavudine	4.057877361	Other	Other Pharmacology	Antivirals
5136	Rizatriptan benzoate	5.686443479	Neuroscience	7-TM Receptors	5-HT1B Receptors
5139	Telmisartan	ND	Cardiovascular	7-TM Receptors	Angiotensin AT1 Receptors
5181	Tolvaptan	23.35544174	Cardiovascular	7-TM Receptors	Vasopressin Receptors
5250	Ramipril	5.060651735	Cardiovascular	Enzymes	Angiotensin-Converting Enzyme
5263	Bazedoxifene acetate	ND	Cancer	Nuclear Receptors	Estrogen and Related Receptors
5359	Rifaximin	16.74287008	Cancer	Nuclear Receptors	Pregnane X Receptor
5491	Aliskiren hemifumarate	3.240232754	Endocrinology	Enzymes	Other Proteases
5584	Aripiprazole	ND	Neuroscience	7-TM Receptors	D2 Receptors
5798	Irbesartan	5.89918691	Cardiovascular	7-TM Receptors	Angiotensin AT1 Receptors
5828	Ambrisentan	2.815660461	Cardiovascular	7-TM Receptors	Endothelin ETA Receptors
5856	Ritonavir	0.051657733	Endocrinology	Enzymes	Other Proteases
5906	Imatinib mesylate	0.097640396	Cancer	Enzymes	Abl Kinase
6176	FTY 720	ND	Immunology	7-TM Receptors	Sphingosine-1-phosphate Receptors
6185	Pemetrexed	8.645668112	Cancer	Enzymes	Thymidylate Synthetase
6219	Arformoterol tartrate	16.90734954	Cardiovascular	7-TM Receptors	Adrenergic Beta-2 Receptors
6230	Rucaparib camsylate	105.7499442	Cancer	Enzymes	Poly(ADP-ribose) Polymerase
6232	Bosentan	1.376367043	Cardiovascular	7-TM Receptors	Non-selective Endothelin
6234	Entecavir	9.71081886	Immunology	Enzymes	RNA/DNA Polymerase
6285	Megestrol Acetate	13.05811736	Cancer	Nuclear Receptors	Progesterone Receptor
6305	Lenalidomide	7.162168578	Cancer	Enzymes	Ubiquitin E3 Ligases
6311	Tadalafil	39.30212055	Immunology	Enzymes	Phosphodiesterases
6336	Trazodone hydrochloride	53.40198368	Neuroscience	7-TM Receptors	5-HT2A Receptors
6343	Rosuvastatin calcium	0.545552946	Cardiovascular	Enzymes	HMG-CoA Reductase
6357	Ciclesonide	ND	Immunology	Nuclear Receptors	Glucocorticoid Receptor
6384	Ciclopirox	ND	Cancer	Enzymes	Histone Demethylases
6431	Phentolamine Mesylate	15.60940227	Neuroscience	7-TM Receptors	Non-selective Adrenergic Alpha Receptors
6486	Aprepitant	ND	Neuroscience	7-TM Receptors	NK1 Receptor
6641	Roflumilast	15.72603638	Immunology	Enzymes	Phosphodiesterases
6663	Silodosin	27.51926323	Neuroscience	7-TM Receptors	Adrenergic Alpha-1 Receptors
6793	Dasatinib	ND	Cancer	Enzymes	Src Kinases
6811	Lapatinib	ND	Cancer	Enzyme-Linked Receptors	EGFR
6814	Sorafenib	ND	Cancer	Enzymes	Raf Kinase
6819	Mefloquine hydrochloride	ND	Neuroscience	Cell biology	Gap Channels
