# Supplementary figures and images for: Mirtazapine Induces Lipocalin-Type Prostaglandin D Synthase Expression in Brain Pericytes
Source: Biomolecules. 2026 Jun 24;16(7):945. doi: 10.3390/biom16070945 (PMC13407077; doi:10.3390/biom16070945)

**Figure 2I**

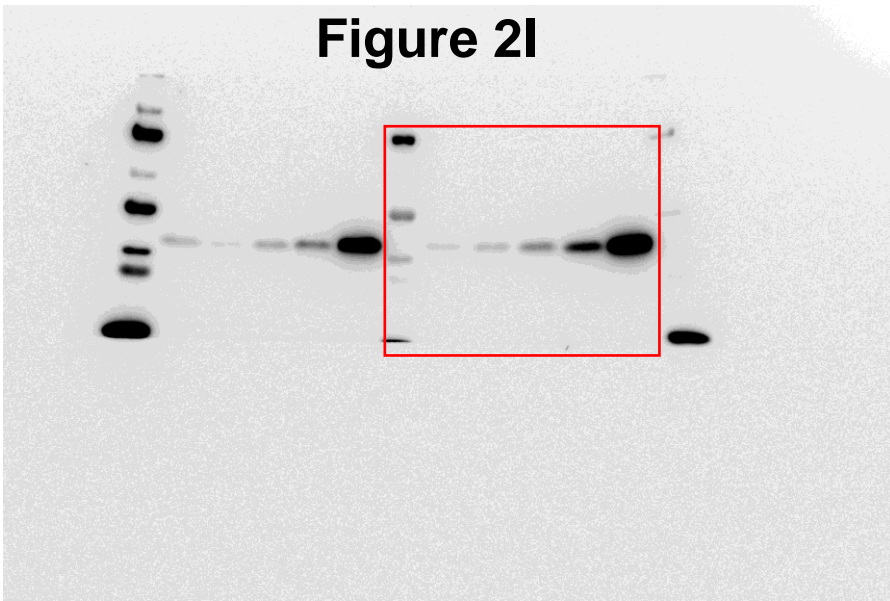

**Figure 2K**

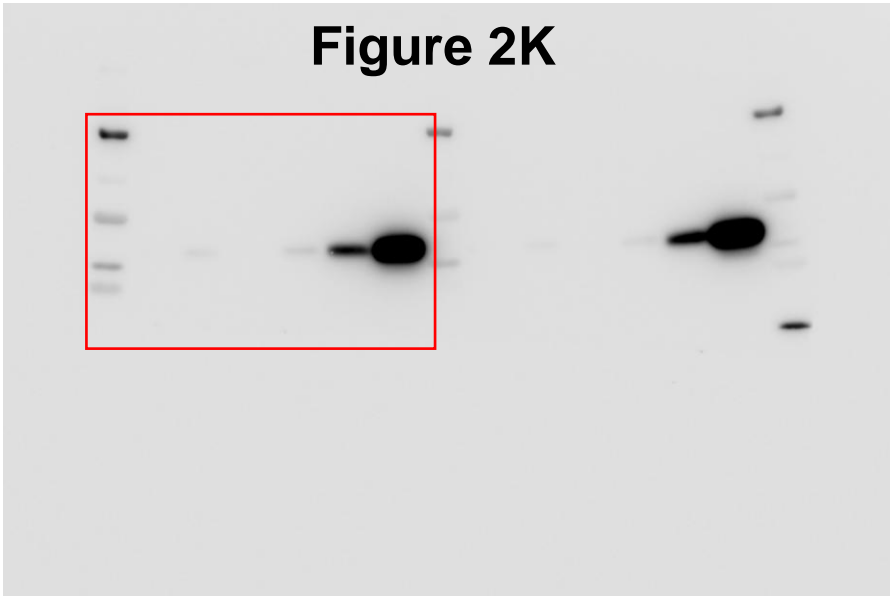

**Figure 6G**

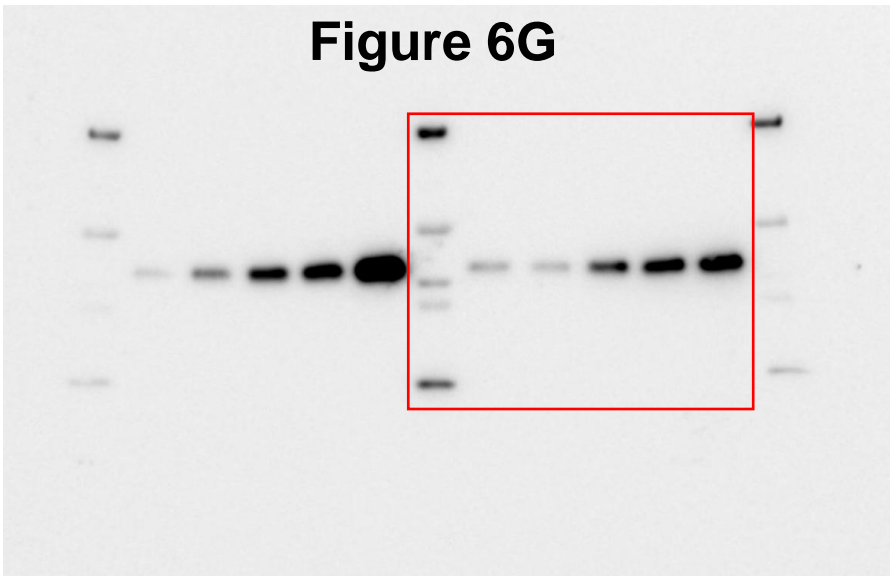

Supplement: Supplementary file 1 [file biomolecules-16-00945-s001.zip › biomolecules-4392781-original-images.pdf]
